# The standardized herbal formula, PM014, ameliorated cigarette smoke-induced lung inflammation in a murine model of chronic obstructive pulmonary disease

**DOI:** 10.1186/1472-6882-13-219

**Published:** 2013-09-05

**Authors:** Kyung-Hwa Jung, Kyoung-Keun Haam, Soojin Park, Youngeun Kim, Seung Ryel Lee, Geunhyeog Lee, Miran Kim, Moochang Hong, Minkyu Shin, Sungki Jung, Hyunsu Bae

**Affiliations:** 1 Department of Physiology, College of Korean Medicine, Kyung Hee University, #1 Hoekidong, Dongdaemoonku, Seoul 130-701, Republic of Korea; 2 Central Research Institute, Hanlim Pharm. Co. Ltd., 1007 Yoobang Dong, Yongin, Kyounggi Do, Republic of Korea; 3 Division of Allergy and Respiratory System, Department of Internal Medicine, College of Korean Medicine, Kyung Hee University, #1 Hoekidong, Dongdaemoonku, Seoul 130-701, Republic of Korea

**Keywords:** COPD, CS, PM014, Neutrophil, IL-6, TNF-α, MCP-1

## Abstract

**Background:**

In this study, we evaluated the anti-inflammatory effect of PM014 on cigarette smoke induced lung disease in the murine animal model of chronic obstructive pulmonary disease (COPD).

**Methods:**

Mice were exposed to cigarette smoke (CS) for 2 weeks to induce COPD-like lung inflammation. Two hours prior to cigarette smoke exposure, the treatment group was administered PM014 via an oral injection. To investigate the effects of PM014, we assessed PM014 functions *in vivo*, including immune cell infiltration, cytokine profiles in bronchoalveolar lavage (BAL) fluid and histopathological changes in the lung. The efficacy of PM014 was compared with that of the recently developed anti-COPD drug, roflumilast.

**Results:**

PM014 substantially inhibited immune cell infiltration (neutrophils, macrophages, and lymphocytes) into the airway. In addition, IL-6, TNF-α and MCP-1 were decreased in the BAL fluid of PM014-treated mice compared to cigarette smoke stimulated mice. These changes were more prominent than roflumilast treated mice. The expression of PAS-positive cells in the bronchial layer was also significantly reduced in both PM014 and roflumilast treated mice.

**Conclusions:**

These data suggest that PM014 exerts strong therapeutic effects against CS induced, COPD-like lung inflammation. Therefore, this herbal medicine may represent a novel therapeutic agent for lung inflammation in general, as well as a specific agent for COPD treatment.

## Background

Chronic obstructive pulmonary disease (COPD) is a common public health concern worldwide, and the incidence of COPD is increasing globally [1,2]. COPD is characterized by progressive and irreversible airway obstruction [3]. Chronic inflammation contributes to a decline in pulmonary function associated with chronic bronchitis, mucus hypersecretion, and emphysema via the release of pro-inflammatory mediators, reactive oxygen species, and tissue degradation enzymes [4]. Subsequently, these pulmonary changes result in abnormalities in gas exchange at the pulmonary level and respiratory failure [5]. The pathology of COPD differs markedly from that of asthma [6-8]. In larger airways, there is evidence of neutrophilic rather than eosinophilic inflammation, as indicated by an increased number of neutrophils in BAL fluid. Current conventional treatment is aimed at relieving symptoms, preventing recurrent exacerbation, preserving optimal lung function and enhancing overall quality of life [9]. Although many drugs are used to treat COPD, the adverse effects associated with several classes of drugs, such as steroids, have increased the need for alternative treatments, such as herbal medicines [10,11].

Because COPD is a chronic inflammatory disorder, it is essential to determine whether novel anti-inflammatory agents can halt or slow the decline in lung function that occurs in response to this disease when selecting candidate drugs. Several studies have demonstrated that compounds derived from plants have anti-inflammatory or immune-modulating properties [12], and several herbal medicines, including *Panax ginseng* and *Salvia miltiorrhiza,* have been used for COPD treatment [13].

PM014 is modified from Chung-Sang-Bo-Ha-Tang (CSBHT). The Chung-Sang-Bo-Ha-Tang (CSBHT) has been especially used to treat chronic pulmonary diseases in Korea for centuries [14]. Previously, we developed the formulation of PM014 based on the series of *in vitro* and *in vivo* screening efforts. The results showed that PM014 possessed potent anti-inflammatory effects in both lipopolysaccharide (LPS)-induced and elastase/LPS induced acute lung inflammation murine models [15]. However, the previous study only suggested the prophylactic effects of PM014 in lung injury. In addition, the lung inflammation inducer did not reflect the actual environment. To overcome these limitations, we evaluated PM014 to determine if it had therapeutic effects on lung inflammation in a mouse model of CS-induced lung neutrophilia that mimicked a COPD-like lung injury [16]. CS-associated chronic obstructive pulmonary disease (COPD) is characterized by inflammation, changes affecting small airways and the development of emphysema [17]. The main pathological characteristics of CS-associated COPD are inflammation along the bronchus and bronchioles, fibrosis, smooth muscle hypertrophy, goblet cell hyperplasia, small airway and vascular remodeling, and development of centrilobular emphysema [18]. In addition, CS is a very strong environmental risk factor that is linked to rheumatoid arthritis (RA) [19,20] and other autoimmune diseases [21-23]. CS is a toxic and carcinogenic mixture of more than 5,000 chemicals [24]. Of these chemicals, approximately 400 have been quantified; at least 200 are toxic to humans and/or experimental animals; and over 50 have been identified as known, probable, or possible human carcinogens [25]. Mainstream smoke typically contains large amounts of bacterial and fungal compounds, such as endotoxins (lipopolysaccharide, LPS, in the outer membrane of Gram-negative bacteria) and ergosterol (a specific fungal membrane lipid) [26].

Roflumilast is indicated as a treatment to reduce the risk of COPD exacerbations in patients with severe COPD associated with chronic bronchitis and a history of exacerbation [27]. Roflumilast is a lipophilic, highly permeable molecule that exhibits rapid and nearly complete absorption after oral administration [28].

In the present study, we evaluated the anti-inflammatory effects of PM014 on CS-induced lung inflammation in mice and elucidated the possible mechanism by which PM014 suppresses CS-induced lung inflammation.

## Methods

### Reagent

PM014, contains 7 species of medicinal plants, was purchased from Kyung Hee Herb Pharm (Seoul, South Korea) and processed in Hanlim Pharm Co. LTD (Seoul, South Korea). Every herb of PM014 was cut and mixed amount of 2,100 g as the ratio indicated in Table 1. It was extracted with purified water (2,100 mL) using a reflux for 3 hours at 90 ~ 100°C, and then filtered by using 25 μm sieve. Supernatant was concentrated at 60°C under vacuum using an evaporative system. Extracts and 260 g of dry corn starch were mixed, and then vacuum dried at 60°C. The PM014 extract powder was dissolved in PBS. The amount of standard materials in the final extracts (1 g) of PM014 were; Paeoniflorin > 0.43 mg, Schizandrin > 0.12 mg, Baicalin > 7.26 mg, and Amygdalin > 2.48 mg. Roflumilast (Santa Cruz Biotechnology, Inc. Delaware Avenue, CA, U.S.A.), which was used as a positive control, was also dissolved in PBS.

**Table 1:** Composition and amount of PM014

**Herb**	**Pharmaceutical name**	**Amount (g)**
Suckjihwag	Rehmannia Radix Preparata	600
Mockdanpi	Moutan Cortex	300
Omija	Schisandrae Fructus	300
Chunmundong	Asparagi Tuber	300
Hengin	Armeniacae Semen	225
Hwangkum	Scutellariae Radix	225
Baekbukuen	Stemomae Radix	150
	Total	2,100 g

### Animal and maintenance conditions

**Figure 1:**
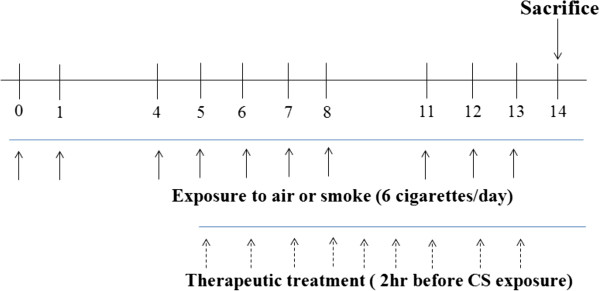
**Schematic diagram of the experimental protocol.** Specific pathogen-free, 7-week-old, female balb/c mice were administered cigarettes on days 0 to 1, 4 to 8, and 11 to 13, as outlined above in the general exposure procedures. The mice were sacrificed on day 14.

In this study, female Balb/c (6–7 weeks of age) mice (n= 5–6 per group) were whole-body exposed to room fresh air or cigarette smoke of 6 cigarettes (Reference Cigarette to 3R4F without a filter, University of Kentucky, Lexington, KY, U.S.A.) a day for 2 weeks, one cigarette every 5 min for total exposure time 30 min (cigarette was completely burned in the first 1 min). The mice were exposed to cigarettes in smoke chamber (1.54 m × 0.52 m × 0.22 m, Live Cell Instrument, Seoul, South Korea) from on days 0 to 1, 4 to 8, and 11 to 13 as outlined in overall exposure procedures (Figure 1). For treatment, each group of mice were orally administered 2 hr before cigarette smoke with CS group, rolfulmilast (ROF) group, as a positive control group, and mixtures of PM014 (50, 100, 200 mg/kg wt) groups for the duration of the study from day 5 to 13. The control (CON) group (age and sex-matched Balb/c mice) were exposed to fresh air instead of CS. Twenty-four hours after the last exposure, mice were sacrificed and collect BAL fluid and lung specimen. These experiments were performed twice. The experimental procedure was approved by the Institutional Animal Care and Use Board of Kyung Hee University (KHUASP (SE)-12-015).

### Analysis of BAL cells

The mice were sacrificed via cervical vertebral dislocation. PBS (phosphate buffered saline) was slowly infused into the lungs and withdrawn via a cannula inserted into the trachea. The cell numbers were counted using a hemocytometer, and differential cell counts were performed on slides prepared by cytocentrifugation at 250 rpm for 3 min and Diff-Quick staining. Approximately 500 cells were counted. BAL fluid was then centrifuged, and the supernatants were kept at −80°C.

### ELISA measurements of IL-6 and TNF-α and MCP-1 in bronchoalveolar lavage fluids

Protein concentrations were determined using a BCA kit (Pierce Biotechnology Inc., Rockford, IL, U.S.A.). IL-6, TNF-α, and MCP-1 concentrations were measured with a quantitative sandwich enzyme-linked immunoassay kit (BD, San Diego, CA, U.S.A.). A 96-well microtiter plate was incubated overnight at 4°C with anti-rat IL-6,TNF-α and MCP-1 monoclonal antibody in coating buffer, washed with PBS containing 0.05% tween 20 (Sigma, St. Louis, MO, U.S.A.) and blocked with 5% FBS in PBS for 1 hr at room temperature. Subsequently, the BAL fluid (100 μl) was incubated for 2 hr at room temperature. Then, secondary peroxidase-labeled biotinylated anti-rat IL-6, TNF-α, and MCP-1 monoclonal antibody was incubated in 5% FBS in PBS for 1 hr. Finally, the plates were treated with TMB substrate solution for 30 min, and the reaction was stopped by adding TMB stop solution (BD, San Diego, CA, U.S.A.). Optical density was measured at 450 nm in a microplate reader (SOFT max PRO software, Sunnyvale, CA, U.S.A.).

### Preparation of lung tissues and histology

The lung tissues were removed from the mice, and the right lower lobes were removed for histological analysis. Four percent paraformaldehyde fixing solution was infused into the lungs. The specimens were dehydrated and embedded in paraffin. For histological examination, 4 μm sections of embedded tissue were cut on a rotary microtome, placed on glass slides, deparaffinized, and stained sequentially with hematoxylin and eosin (H&E). The severity of peribronchial inflammation was graded semi-quantitatively as previously described [29]. Hyperplasia of the goblet cells within the bronchial epithelium was assessed by counting cells in periodic acid Schiff (PAS)-stained sections. Slides were mounted with Canada balsam (Showa Chemical Co. Ltd., Tokyo, Japan). PAS-positive cells in the epithelium and total epithelial cells were counted, and the percentage of PAS-positive cells was calculated. For quantitating air space in lung, the sections with the maximum parenchyma cross-sections were selected for morphometric analysis using a digitized image tool [30]. Micrographs were obtained using Image Pro-Plus 5.1 software (Media Cybernetics, Inc. Silver Spring, MD, U.S.A.).

### Statistical analysis

Data are presented as the means ± S.E.M. Data analysis was conducted using Graphpad Prism software (version 4, San Diego, CA, U.S.A.). The differences between study groups were determined by one-way ANOVA and the Newman-Keuls multiple comparison test. *P* < 0.05 was considered to be statistically significant.

## Results

### The effects of PM014 on total and inflammatory cells levels in BAL fluid

**Figure 2:**
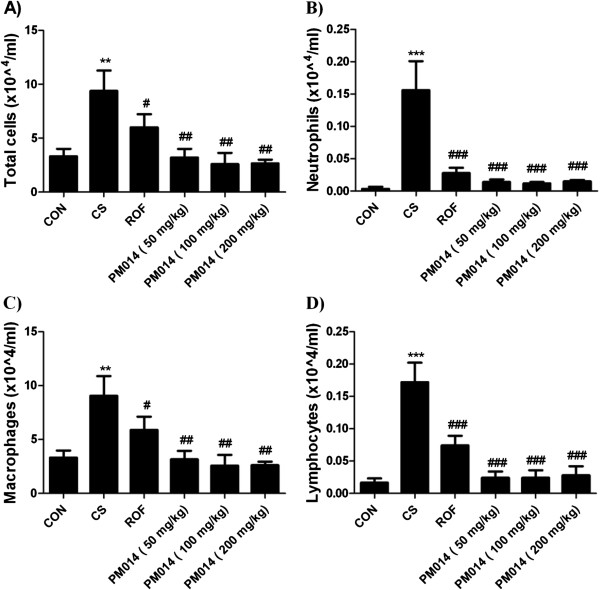
**Cellular profiles in BAL fluid and microscopic findings of the intrapulmonary bronchi.** After sacrificing the mice, PBS buffer was slowly infused into the lungs and withdrawn via a cannula inserted into the trachea. The cell numbers were counted using a hemacytometer, and differential cell counts were performed on slides prepared by cytocentrifugation at 250 rpm for 3 min and Diff-Quick staining. **A)** Count of total cell number, **B)** Count of neutrophils, **C)** Count of macrophages, **D)** Count of lymphocytes. Data are shown as mean ± S.E.M. Statistical analyses were conducted by one-way ANOVA followed by Newman-Keuls Multiple Comparison test (***p < 0.001, **p < 0.01 vs. CON, ###*p* < 0.001, ##*p* < 0.01, #*p* < 0.05 vs. CS; n = 5–6).

We made an experiment with various doses of PM014 (0.1, 1, 10 and 100 mg/kg wt) to test its anti-inflammatory effect on CS-induced COPD. As a result, PM014 did not show significant effect on concentration of 10 mg/kg and below, however, dose of 100 mg/kg significantly decreased number of total leukocytes, neutrophils, macrophages and lymphocytes in BAL fluid compared with CS-exposure group (Table 2). To find the optimal dosage of PM014, we conducted separate experiment with three different dosages of PM014 around 100 mg/kg (50, 100 and 200 mg/kg wt). Cigarette smoke exposed (CS) group showed significantly increased numbers of neutrophils, lymphocytes, macrophages and total cells compared with fresh air exposed (CON) group. Treatment groups with PM014 (50, 100, 200 mg/kg wt) or roflumilast (ROF) group exhibited remarkably decreased numbers of total cells, neutrophils, lymphocytes and macrophages in BAL fluid compared with the CS-exposure group (Figure 2).

**Table 2:** Bronchoalveolar analysis

				**PM014**	**PM014**	**PM014**	**PM014**
**(× 10**^**4**^**/ml)**	**CON**	**CS**	**ROF**	**0.1 mg/kg**	**1 mg/kg**	**10 mg/kg**	**100 mg/kg**
**Total cells**	2.80 ± 1.10	12.00 ± 1.79^***^	6.20 ± 1.10^#^	11.60 ± 2.97	11.20 ± 3.90	10.40 ± 4.78	5.67 ± 2.66^##^
**Neutrophils**	0.00 ± 0.00	0.31 ± 0.02^***^	0.08 ± 0.03^##^	0.28 ± 0.11	0.26 ± 0.12	0.19 ± 0.05	0.06 ± 0.07^###^
**Macrophages**	2.78 ± 1.08	12.30 ± 1.21^***^	6.37 ± 1.20^##^	11.20 ± 2.85	11.17 ± 4.41	8.24 ± 2.40	4.70 ± 2.29^###^
**Lymphocytes**	0.01 ± 0.02	0.17 ± 0.03^***^	0.09 ± 0.04^#^	0.12 ± 0.05	0.13 ± 0.04	0.11 ± 0.06	0.03 ± 0.02^###^

Data are shown as mean ± S.E.M. *CON* control group, **CS** cigarette smoke exposed group, and *ROF* roflumilast treated group. Statistical analyses were conducted by one-way ANOVA followed by Newman-Keuls Multiple Comparison test (***p < 0.001 vs. CON, ###*p* < 0.001, ##*p* < 0.01, #*p* < 0.05 vs. CS; n = 4–6).

### The effect of PM014 on IL-6, TNF-α and MCP-1 in BAL fluids by CS-induced mice

**Figure 3:**
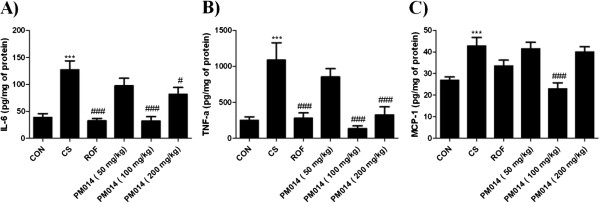
**The effect of PM014 on IL-6, TNF-α and MCP-1 in BAL fluid.** BAL fluids were collected as described in Figure 2. **A)** Level of IL-6, **B)** Level of TNF-α, **C)** Level of MCP-1 concentrations were measured with a quantitative sandwich enzyme-linked immunoassay. Data are shown as mean ± S.E.M. Statistical analyses were conducted by one-way ANOVA followed by Newman-Keuls Multiple Comparison test (***p < 0.001 vs. CON, ###*p* < 0.001, #*p* < 0.05 vs. CS; n = 5–6).

Pro-inflammatory cytokines (TNF-α, IL-6) contribute to cigarette smoke induced COPD. MCP-1 is potent chemoattractant of monocytes and acts on the recruitment of macrophages in COPD. To evaluate the anti-inflammatory effects of PM014, the secretion of pro-inflammatory cytokines and CC chemokine in BAL fluid were measured. Secretion of TNF-α, IL-6 and MCP-1 were significantly elevated in CS group when compared with the CON group. Positive treatment with ROF group showed markedly reduced levels of IL-6 and TNF-α than CS group. However, ROF group failed to inhibit MCP-1 releasing. The PM014 (100, 200 mg/kg wt) groups also showed reduced level of IL-6 and TNF-α in BAL fluid than CS group. Decrement of MCP-1 was only observed 100 mg/kg treatment of PM014 compared with CS group. In addition, PM014 (100 mg/kg wt) group was similar to the CON group on IL-6, TNF-α and MCP-1. However, treatment with PM014 (50 mg/kg wt) data not shown significantly decreased of IL-6, TNF-α and MCP-1 levels than CS group (Figure 3).

### The Effect of PM014 on histologic lung damage

**Figure 4:**
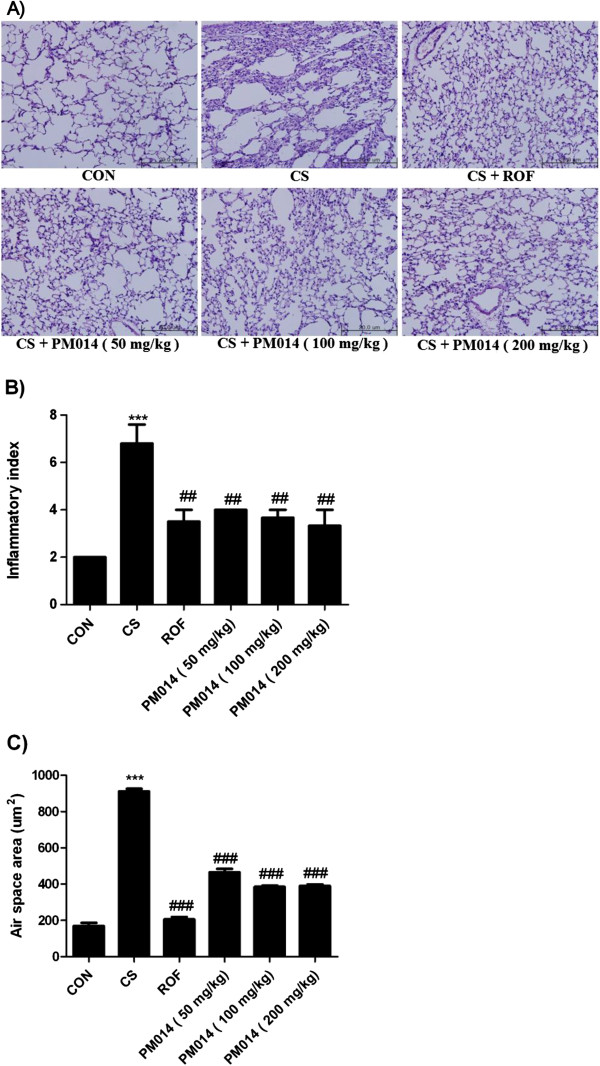
**The effect of PM014 morphology changes in CS exposure mice. A)** Right lower lobes of mice were dissected and stained with hematoxylin and eosin. **B)** The degree of inflammation was quantified using a semi-quantitative scale. **C)** Air space was calculated by Image Pro-Plus 5.1 software as described in the Materials and Methods section. Data are shown as mean ± S.E.M. Statistical analyses were conducted by one-way ANOVA followed by Newman-Keuls Multiple Comparison test (***p < 0.001 vs. CON, ##*p* < 0.01 vs. CS).

To determine if PM014 exerted an effect on CS-induced lung damage, lung sections were stained with hematoxylin and eosin (H&E). The results of histological examination of lung tissue paralleled the cell number in the BAL fluid. Marked influxes of inflammatory cells into the peribronchial layer and intraluminal areas were detected in the lung sections of CS group. Additionally, PM014 treatment led to a marked reduction in the infiltration of inflammatory cells within the lung in CS group that was similar to the results observed in ROF group. These results demonstrated that treatment with PM014 inhibited CS-induced inflammation in lung tissue in a fashion similar to ROF group (Figure 4A, 4B). In addition, CS exposure in mice led to airspace enlargement. Performing qrofuantitative analysis of alveolar airspace enlargement, CS group showed alveolar destruction, which resulted in enlarged air spaces, indicating emphysematous change than in the that treatment with PM014 groups. In contrast, mice treated with PM014 showed less alveolar damage (Figure 4C).

### The effect of PM014 on goblet cell hyperplasia in bronchial airways

**Figure 5:**
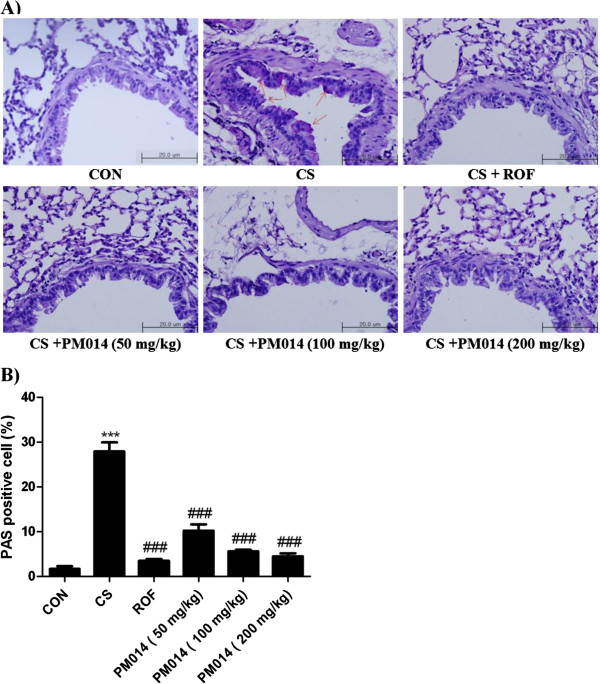
**The effect of PM014 on goblet cell number.** The right lower lobes of mice were dissected and stained with periodic acid Schiff. **A)** The arrow indicates the PAS-positive cells. **B)** Periodic acid Schiff (PAS)-positive mucosal goblet cells around the bronchial airway were counted and are depicted as the percentage of goblet cells, as described in the Materials and Methods section. Data are shown as mean ± S.E.M. Statistical analyses were conducted by one-way ANOVA followed by Newman-Keuls Multiple Comparison test (***p < 0.001vs. CON, ###*p* < 0.001 vs. CS).

To evaluate the effect of treatment with PM014 in goblet cell hyperplasia, lung tissues were stained using periodic acid-Schiff (PAS) (Figure 5A). Consistent with previous results, PAS-positive mucus-counting goblet cells around the bronchial airway epithelium of were more abundantly detected in the CS group than CON group. On the other hand, PM014 treatment considerably decreased PAS-positive goblet cells around the bronchial airway epithelium (Figure 5B). Taken together, these findings indicate that treatment with PM014 has a powerful therapeutic effect on CS-induced lung inflammation.

## Discussion

Inflammation in COPD is complicated, with inflammatory and structural cells that release various mediators, including mediators such as LTB_4_, IL-8 and GCP-2, which were chemoattractant for neutrophil and chemokines such as MCP-1 and MIP-1α, which attract macrophage [31]. An accumulation of inflammatory cells such as neutrophils, macrophages, dendritic cells CD+8T lymphocytes is seen [32]. In our study, the infiltration of inflammatory cells in BAL fluid and in the lung parenchyma was observed following exposure to cigarettes. Neutrophils have been implicated in causing tissue damage in COPD through the release of a number of mediators, including proteases, such as elastases and matrix metalloproteinase, and oxidants and toxic peptides, such as defensins [33].

Concomitant with the influx of neutrophils, increased levels of the pro-inflammatory cytokines, TNF-α and IL-6 were observed in the BAL fluid by Cigarette smoke (CS) exposure induce mice model. In addition, the expression of inflammation-related cytokines, such as IL-6 and TNF-α was found to increase following CS exposure in the airway. Also, CS-induced goblet cell hyperplasia is associated with the development of bronchitis which is related to COPD, which depends on the degree of epithelial inflammation [34]. Goblet cell hyperplasia is one of the morphogic changes in lung epithelium. In addition to that, there are various epithelial changes including submucosal gland hypertrophy associated with loss of ciliated epithelial cell number, leading to reduced mucociliary clearance, and mucus plug formation [35].

Cigarette smoke can contribute to the development of many human diseases, such as cardiovascular disease, lung cancer, asthma, and chronic obstructive pulmonary disease. Thousands of compounds are present in CS, including a large number of reactive oxygen species that can cause DNA damage and lead to the activation of poly (ADP-ribose) polymerase (PARP) enzyme [36]. Other components of CS, such as nicotine and acrolein, have also been shown to exert direct genotoxic effects [37,38].

In our study, the infiltration of inflammatory cells in BAL fluid was observed after CS exposure. Exposure to CS induced several pathological changes, such as inflammatory cell accumulation in the lung parenchyma, hyperplasia of goblet cells, hypersecretion of mucus, alveoli enlargement, and increases in collagenic and elastic structures in the alveolus. The pathological changes observed in this study were very similar to the clinical features of COPD patients [33]. In this CS exposure model, the herbal mixture PM014 showed consistent efficacy comparable with that of the commercially available anti-COPD drug, roflumilast [39].

Herbal mixtures are widely used as traditional medicines to treat many different types of disease [40,41]. In Korean traditional medicine, herbs are used as mixtures rather than as one herb by itself. PM014 is modified from Chung-Sang-Bo-Ha-Tang (CSBHT). The Chung-Sang-Bo-Ha-Tang (CSBHT) has been especially used to treat chronic pulmonary diseases in Korea for centuries. However, CSBHT contains 18 species of medicinal plants, and it is difficult to standardize the herbal formula [14]. Therefore, CSBHT was modified to PM014, which contains 7 species of medicinal plants. Previously, we initially compared the effects of each herb and the PM014 herbal mixture in an acute LPS-induced lung injury model [15]. *Stemona sessilifolia, S. japonica* and *S. tuberose* are the three original sources of Stemonae Radix specified in Chinese Pharmacopoeia (CP) and have been traditionally used as antitussive and insecticidal remedies [42]. *Asparagus cochinchinensis* is used for treating lung- and spleen-related diseases [43]. A bioactive flavonoid extracted from the root of *Scutellaria baicalensis* has anti-inflammatory and anti-angiogenic activities [44]. *Schisandra chinensis* fruit especially alleviates cough and satisfies thirst. In modern pharmaceutical studies, *Schisandra chinensis* fruit has been reported to reduce hepatotoxicity [45-50]. The root of *Rehmannia glutinosa* (RR) is commonly used to reduce inflammation [51]. The root cortex of Paeonia suffruticosa Andrews (PSA), also known as Moutan Cortex, is known to have anti-allergic and anti-inflammatory properties [52]. Individual herb extracts in PM014 also attenuated the immune cell influx; however, treatment with the herbal mixture PM014 resulted in less recruitment of all immune cells toward the lungs than the individual herbal treatments [15]. Therefore, it is assumed that using a mixture of 7 medicinal herbs is more effective with regard to synergism than using each of the 7 herbs separately. These results may suggest that PM014 is a powerful therapeutic agent, which reduces chronic accumulation of inflammatory cells induced by CS. In this study, PM014 (50, 100, 200 mg/kg wt) markedly reduced inflammatory cells in BAL fluid. Furthermore, when we compared the histological findings, PM014 (100 mg/kg wt) significantly reduced the numbers of lymphocytes, neutrophils, infiltrating macrophage and the level of goblet cell metaplasia. Epithelial basement membrane thickening and inflammation of the bronchiole also were remarkably inhibited. PM014 (100 mg/kg wt) also, markedly reduced levels of TNF-α, IL-6 and MCP-1 than CS group. However, PM014 (50 mg/kg wt) did not show significant effect than PM014 (100, 200 mg/kg wt) in TNF-α, IL-6, and MCP-1 production.

TNF-α, an early pro-inflammatory cytokine, is believed to trigger the activation of other pro-inflammatory cytokines, such as IL-6 and IL-8 [53]. TNF-α also activates nuclear factor-κB, which increases IL-8 gene transcription, thereby inducing the release of IL-8 from the airway epithelium and neutrophils. IL-8, a CXC chemokine, is a neutrophil chemoattractant and activator [33]. MCP-1 a monocyte selective chemokine which attracts monocytes to lung is increased in lung COPD patients [54]. Macrophages mediate inflammation in COPD through the release of chemokines that attract neutrophils, monocytes and T-cells and releases serine proteases like matrix metalloproteinase (MMP-9) [55].

In our study, PM014 downregulated pro-inflammatory cytokine production in both acute and chronic lung inflammation. Therefore, treatment with PM014 may act steadily on downstream events, including the influx of inflammatory cells and the levels of mediators aggravating the inflammatory response. In addition, histopathological data implied that PM014 administration inhibited the progression of airspace enlargement and goblet cell hyperplasia. Theses result also demonstrated that PM014 could be sufficient to structural changes of lung, typical of COPD.

## Conclusions

The results of this study provide evidence that treatment with PM014 exerts therapeutic effects against smoking-induced lung inflammation in mice. The remarkable anti-inflammatory effects exerted by PM014 suggest that it has the potential to be used in the treatment of COPD patients. However, further study to elucidate the mechanisms underlying the action of PM014 should be conducted to aid in the discovery of new therapeutic agents for COPD treatment.

## Competing interests

The authors declared that they have no competing interest.

## Authors’ contribution

KHJ, KKH, SP, SRL and GL have made contribution to acquisition and analyzing data. MK, MH and MS have made been involved in interpretation of data. YK, SJ and HB have been involved in designing the study and drafting the manuscript. All authors read and gave final approval for the version submitted for publication.

## Pre-publication history

The pre-publication history for this paper can be accessed here:

http://www.biomedcentral.com/1472-6882/13/219/prepub
